# Serum and bone pentosidine in patients with low impact hip fractures and in patients with advanced osteoarthritis

**DOI:** 10.1186/s12891-016-1168-7

**Published:** 2016-07-22

**Authors:** Jan Vaculík, Martin Braun, Pavel Dungl, Karel Pavelka, Jan J. Stepan

**Affiliations:** Department of Orthopedics, Faculty of Medicine 1, Charles University Prague, and Bulovka Hospital, Prague, Czech Republic; Department of Composites and Carbon Materials, Institute of Rock Structure and Mechanics, Academy of Sciences of the Czech Republic, Prague, Czech Republic; Institute of Rheumatology, Prague, and Faculty of Medicine 1, Charles University Prague, Na Slupi 4, Prague, CZ 12850 Czech Republic

**Keywords:** Biomarkers, Osteoarthritis, Osteoporosis, Pentosidine, Proximal femoral fracture

## Abstract

**Background:**

Femoral neck fractures are a common occurrence in patients suffering from osteoporosis, while intracapsular hip fracture is rare in cases of osteoarthritis of the hip. Previous histomorphometric studies have emphasized the association between bone microarchitecture and the risk of low-impact fractures in osteoarthritis and osteoporosis patients. However, the strength of bone material is also a function of composition of organic bone matrix. In order to compare tissue material properties in these two clinical conditions, serum and bone pentosidine, a non-enzymatic collagen crosslinking element, was measured in patients who suffered a low-impact fracture, and in patients with advanced osteoarthritis.

**Methods:**

The patient population consisted of 70 patients who underwent hemiarthroplasty surgery for a femoral neck fracture, and 41 patients with advanced hip joint osteoarthritis without a history of low- impact fracture, who were indicated for total hip joint replacement. Pentosidine content was analyzed in bone samples and in serum obtained from fracture and osteoarthritis patients using high performance liquid chromatography.

**Results:**

Serum and bone concentrations of pentosidine were higher in subjects with hip fractures compared with osteoarthritis after adjustment for age, sex, weight, serum creatinine, and diabetes. A significant positive correlation was found between bone and serum pentosidine in fractured cases. A comparable relationship was also demonstrated for pentosidine levels in serum and bone relative to differentiation of fracture and osteoarthritis cases.

**Conclusions:**

Serum pentosidine can be considered a potential biomarker for identification of subjects with impaired bone quality and bone strength.

## Background

The strength of bone material is a function of bone microarchitecture, and tissue material properties. At the material level, bone strength is determined by the degree of mineralization of basic structural units, micro-damage accumulation, and properties of the organic bone matrix, namely collagen cross-link formation [1, 2]. These are regulated by cellular activities and tissue turnover rate [3]. Impaired enzymatic cross-linking and/or increases in non-enzymatic cross-links in bone collagen have been proposed as a determinant of impaired bone mechanical properties in aging, involutional osteoporosis, and diabetes mellitus [4, 5]. Reduced bone turnover allows the formation of additional collagen crosslinks by non-enzymatic means, resulting in accumulation of non-enzymatic advanced glycation end-products (AGEs) in bone tissue [6, 7]. Bone collagen glycation allows micro-damage in bone to spread more easily, therefore increasing the total crack surface density, making bone tissue more brittle and more likely to fracture [8, 9].

Out of several AGE crosslinks, pentosidine (PEN) has been quantified in bone [10], and its accumulation has been associated with the age-related degradation of bone mechanical properties [11, 12]. PEN is a senescent non-enzymatic cross-link resulting from a spontaneous interaction between arginine and lysine amino acids and free sugars [13, 14]. PEN has been shown to accumulate with age in cortical bone of human femurs [15]. Accumulation of AGEs in bone collagen matrix has been associated with brittleness of collagen fibers and impairment of the mechanical properties of cortical and trabecular bone [11, 12, 16, 17]. Increased content of PEN in cortical and trabecular bone was associated with impairment of bone quality in osteoporotic patients with hip fractures [18], while plasma PEN levels were not increased in patients with osteoarthritis compared with controls [19]. However, serum and bone PEN has not been compared previously in patients with hip osteoarthritis and low-impact hip fractures.

To examine the performance of PEN as a biomarker of impaired quality of organic bone matrix, potentially associated with fracture risks, we compared PEN levels in serum and proximal femoral bone specimens using high performance liquid chromatography (HPLC) in subjects who had undergone hemiarthroplasty for an intracapsular hip fracture and from a group of fracture-free patients with hip joint osteoarthritis.

## Methods

### Study participants

This cohort study was performed in patients followed as part of routine care in the Department of Orthopedics, Bulovka Hospital, Prague, Czech Republic. Serum and femoral head bone specimens were collected from 70 subjects who had undergone hemiarthroplasty for an intracapsular hip fracture. Simultaneously, bone specimens from the iliac crest were obtained in random from 21 patients. The group with hip joint osteoarthritis, which was without a history of low energy fractures, included 41 patients in advanced stages of hip osteoarthritis, who were indicated for total hip joint replacement, 63 % of the atrophic type, 24 % of the hypertrophic type [20]. A complete clinical history, including details of specific common medical conditions (e.g., hypertension, congestive heart failure, heart attack, and stroke, diabetes mellitus), lifestyle risk factors including smoking (current, past, never) and alcohol consumption, detailed personal history of glucocorticoids use (previous or ongoing), fracture history (type and trauma), and physical examination were assessed. Excluded were patients with Paget’s disease of the bone, malignancy, renal osteodystrophy, hyperparathyroidism, active liver disease or clinical jaundice, previous treatment with aminobisphosphonates, denosumab, teriparatide, vitamin D >50,000 IU/week or active vitamin D analogs. Characteristics of the study subjects are shown in Table 1.

**Table 1:** Characteristics of the study population

	Male		Female	
	Fracture	Controls Osteoarthritis	Fracture	Controls Osteoarthritis
Number of subjects	21	25	49	15
Age (years)	77.3 ± 9,1^a^	64.3 ± 10.9	77.3 ± 11.0	68.0 ± 7.9
Height (cm)	174.0 ± 7.0	176.0 ± 5.1	161.9 ± 6.9	162.9 ± 7.5
Weight (kg)	75.9 ± 15.0 ^b^	89.7 ± 11.5	68.7 ± 15.9	79.0 ± 18.0
Prevalent fracture (No.)	2	0	21 ^a^	0
Glucocorticoids (No.)	1	0	6	1
Smoking (No.)	2	2	5	1
Diabetes (No.)	3	6	11	3
Creatinine (μmol/l)	107 ± 33.9	97.6 ± 21.7	90.9 ± 29.1	79.6 ± 9.1
Bone pentosidine (nmol/g dry bone mass) ^c^	2.96 ^a^	1.10	2.57 ^a^	0.93
	0.55;6.80	0.41;2.41	0.75;5.11	0.45;1.98
Serum pentosidine (nmol/l) ^c^	73.6 ^a^	27.1	71.5 ^a^	30.3
	48.4; 417.4	15.46;80.3	31.4;154.6	16.2;108.0

^a^:*p* < 0.001; ^b^:*p* < 0.005; ^c^:Median a 95 % CI

### Preparation of samples

Bone specimens obtained from the femoral head of femoral neck fracture patients and specimens from the femoral head of patients undergoing total hip replacement for hip joint osteoarthritis were obtained from the central part of the femoral head using a trephine, along the femoral neck axis. The samples were taken more than 5 mm away from the osteotomy plane of the total hip arthroplasty to avoid heat damage to the bone specimen. In addition to bone from femoral heads, full thickness bone specimens from the anterior ilium were harvested 2 cm posterior and 2 cm inferior to the anterior superior iliac spine from 21 patients with hip fractures, using a Rochester Bone Biopsy Trephine. The bone was cleaned of adhering soft tissues and marrow. Bone specimens were weighed and dried to a constant weight using a SpeedVac vacuum evaporator. The specimens of dry bone (10 mg) were then hydrolyzed in 2 ml of 6 M hydrochloric acid at 105 °C for 16 h. Collected blood serum samples were stored at −20 °C until analysis.

### Pentosidine assay

Pentosidine was determined by reversed phase HPLC combined with sensitive fluorescent detection, as published previously [21]. Briefly, serum samples were mixed with equal amounts of 35 % HCl and hydrolyzed for 16 h at 105 °C. The bone samples were treated as above. In the next step, the hydrolysate was purified and pre-concentrated using solid phase extraction; the hydrolysate was mixed with a suspension of cellulose and combined with a solution containing n-butyl alcohol and acetic acid. This mixture was then applied to a purification column filled with cellulose. The PEN-containing fraction was then desorbed using 0.05 mol/l HCl, and the extract was evaporated. The residue was reconstituted in a solution containing 0.02 mol/l heptafluorobutyric acid, 0.01 mol/l ammonium sulphate and acetonitrile (ergo mobile phase), and this was applied to a HPLC system (Shimadzu, LC-10ADvp, Kyoto, Japan) equipped with a C18 reversed phase column (Separon SGX C18, 150 x 3 mm). We monitored the emission signal at 385 nm upon excitation at 335 nm. Synthetic PEN was used as a standard. The concentration of PEN was expressed in nmol/l.

The peak area reproducibility involving complete analytical procedure (including sample hydrolysis, drying, reconstitution, and HPLC analysis) in actual samples was 95.6 %. Reproducibility of the HPLC determination itself was 98.8 %. Based on our PEN measurements, limit of detection was 1.76 nmol/l and limit of quantitation 5.87 nmol/l.

### Data analyses and statistical methods

The sample size was estimated to enable detection of a difference in PEN between the two groups of patients. Results are expressed as percentages for categorical variables and as medians (95 % CI) or means (standard deviation, S.D.) for continuous variables. Between-group comparison of data was performed using the independent Student's *t*-test for continuous variables. A linear regression method analyzing the relationship between two variables was used; statistical significance of the correlation was determined by Pearson coefficients. Multiple linear regressions were used to estimate the differences in serum and bone PEN between fracture and osteoarthritis patients after adjusting for the effect of confounders such as age, sex, weight, serum creatinine, and diabetes. Values of *p* < 0.05 were considered significant. Statistical analysis of data was performed using SigmaPlot 10 software (Systat Software Inc, Germany).

## Results

**Fig. 1:**
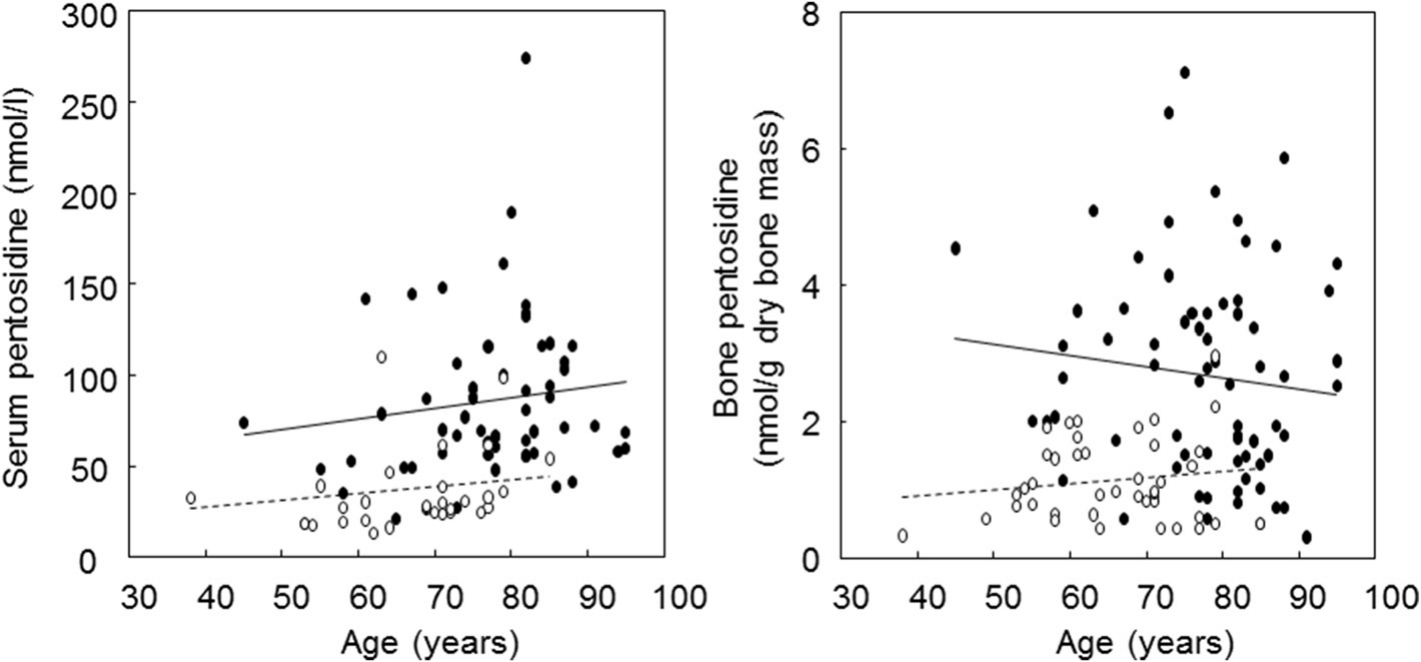
Correlations between age and pentosidine levels in serum and bone in patients with hip fracture (full circles, serum, *r* = 0.13, bone, *r* = 0.11) and osteoarthritis patients (empty circles, serum, *r* = 0.14, bone, *r* = 0.14), n.s. for all

In osteoarthritis cases, a growing trend of serum and bone PEN with age was found. In addition, a growing trend of serum PEN with age was found in individuals with fractures (Fig. 1). In fracture as well as in osteoarthritis patients, no significant correlation was found between serum creatinine and serum or bone PEN. No significant difference was found in PEN content in dry bone (using paired samples) from the femoral head and iliac crest. Serum 25-hydroxyvitamin D was measured in 46 fracture patients, concentrations < 25 nmol/l were found in 22 % of them.

**Fig. 2:**
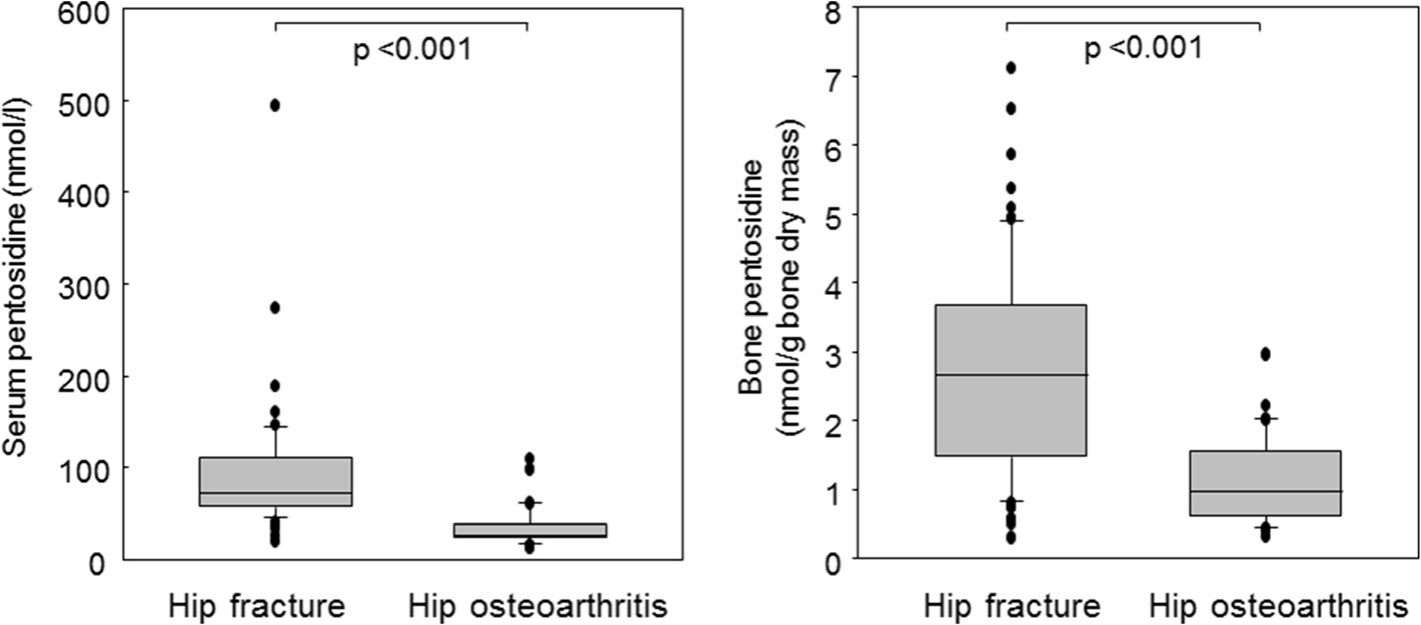
Pentosidine content in serum and bone in patients with hip fracture and in hip osteoarthritis patients

**Fig. 3:**
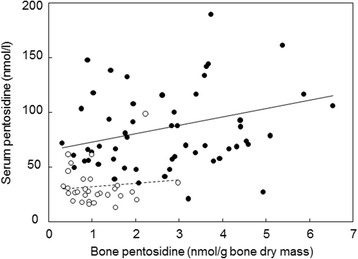
Correlation between bone and serum pentosidine in osteoarthritis patients (empty circles, dotted line, *r* = 0.11, n.s.) and in fractured cases (full circles and line, *r* = 0.27, *p* < 0.05)

**Fig. 4:**
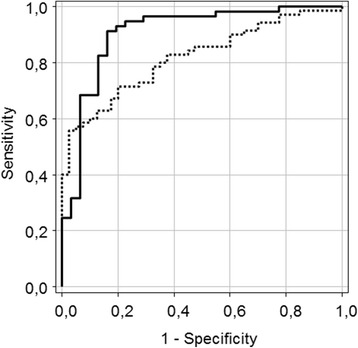
Performance comparison of serum pentosidine, AUC = 0.906 (full line) and bone pentosidine, AUC = 0.845 (dotted line), *p* = 0.274. AUC: area under the curve

Serum and bone concentrations of PEN were significantly higher in subjects with hip fractures compared with osteoarthritis patients (Fig. 2). Significant differences in serum and bone PEN between fracture and osteoarthritis patients remained even after adjustment for age, sex, weight, serum creatinine, and diabetes (*p*  < 0.001). A significant positive correlation was found between bone and serum PEN in fracture patients (Fig. 3). Receiver Operating Characteristics (ROC) curves indicated a comparable performance of serum and bone PEN in differentiation between fracture cases and osteoarthritis patients (Fig. 4).

## Discussion

Previous studies have demonstrated that histomorphometric indices of trabecular bone micro-architecture were more favorable in patients undergoing arthroplasty for hip osteoarthritis than in patients with hip fracture, as well as in comparison with age-matched subjects [22–25]. In this study, bone PEN levels, considered surrogate markers of quality of organic bone matrix, were measured to assess another determinant of strength of bone, the bone tissue material properties. To our knowledge, this is the first study comparing serum and bone pentosidine levels in patients with low-impact hip fractures and in patients with advanced osteoarthritis. Our patients with hip fractures had higher serum and bone PEN concentrations than those with advanced stages of hip osteoarthritis. Previously, PEN content in cortical and trabecular bone of patients with femoral neck fractures has been shown to be higher than in those of age-matched controls [18, 26]. However, the proof of the association between the risk of hip fractures and serum and bone PEN levels would require further studies, considering also bone microarchitecture, bone mineral density (BMD), clinical factors of risk of fracture and namely the general frailty of hip fracture patients should be considered [27, 28].

The association between bone PEN and fracture risk appears independent of BMD mainly in type-I diabetes [29–31]. Also, using an Enzyme-Linked ImmunoSorbent Assay (ELISA), the association between serum PEN levels and changes in BMD in patients treated with bisphosphonate was not confirmed [32]. However, in Japanese postmenopausal women, only a moderate BMD-independent association between urinary PEN and the incidence of vertebral and non-vertebral fractures was observed [28, 33, 34]. In the French OFELY study, there was a significant association between urinary PEN and the risk of all fractures in 396 healthy postmenopausal women, although, the odds ratio was not significant after adjustment for BMD [35].

This study has several limitations. First, the results in our patients must be interpreted within the context of differences in age and body weight, as well as the admitted risk factors for hip osteoarthritis, hip osteoporosis and fractures, although our results were adjusted for age, sex, weight, serum creatinine, and diabetes. The increasing trend in serum and bone PEN levels with age, which were observed in this study, was in accordance with that seen in other studies using HPLC [10], or ELISA [5, 36, 37]. Second, for ethical reasons, bone PEN levels in our patients were not compared with those in healthy age matched subjects from the general population. There was limited data on the differences between PEN levels in osteoarthritis and in healthy subjects, except for an observation that found no significant differences in plasma PEN levels in 17 patients with osteoarthritis compared with controls [19]. Previously, we reported that in patients with knee osteoarthritis, PEN serum concentrations were increased compared with healthy controls, and correlated with a cartilage destruction marker (cartilage oligomeric matrix protein) in synovial fluid [38]. However, knee osteoarthritis is mostly hypertrophic while in the majority of our patients, hip osteoarthritis was mostly of the atrophic type. Thus, although the interpretation of serum PEN levels in patients with knee and hip osteoarthritis and hip fracture should be done with caution, pentosidine crosslinking can nonetheless be considered as a measure of local bone age. Accordingly, serum PEN levels were higher in osteoporotic patients with long term aminobisphosphonate treatment than in BMD-matched controls [39]. Pentosidine formation is closely linked not only to glycation but also to oxidative processes [40, 41]. Accumulation of advanced non-enzymatic glycation end products in numerous body tissues during the course of aging, in diabetes, and in rheumatoid arthritis has been associated with oxidative stress [13]. It remains unknown whether the increase in AGE modified proteins in patients with chronic inflammation merely represents oxidative stress or they actively participate in bone and joint destruction [19]. Also, it is unclear to what extent serum PEN contributes to bone strength at fractured sites, since bone fractures are strain-controlled and PEN affects the organic matrix, which is better known as a contributor to post-yield deformation of bone rather than to its elastic properties [9]. Third, PEN has been shown to be also secreted from tissues other than bone and it is unclear what proportion of serum PEN levels actually reflects PEN content in bone. Compared with controls, PEN levels are significantly increased not only in aging, osteoporosis, diabetes mellitus, renal disorders, and rheumatoid arthritis [4, 5, 42–45], but have also been reported to correlate with the radiographic severity of lumbar spondylosis without vertebral fractures [46]. Increased serum PEN was an independent risk factor for loss of muscle mass in postmenopausal women with type 2 diabetes [47]. Finally, BMD measurements or histomorphometry of tetracycline labeled bone could not be performed in our patients with incident hip fractures, or those undergoing arthroplasty for hip osteoarthritis. Serum markers such as type 1 collagen cross-linked C-telopeptide (βCTX) and procollagen type I amino-terminal propeptide (PINP) were not measured in our patients, since both markers are strongly affected by incident fractures. These markers reflect whole body rates of bone resorption and bone formation rather than the rates of remodeling at specific skeletal sites containing different ratios of trabecular to cortical component, each with its own metabolic rates [48].

## Conclusions

Serum pentosidine can be considered a potential biomarker for identification of subjects with impaired quality of organic bone matrix. However, more studies are necessary to define the predictive value of serum PEN concerning fractures and the efficacy and safety of bone-active drugs in osteoporosis therapy.

## Abbreviations

AGEs, advanced glycation end-products; BMD, bone mineral density; ELISA, enzyme-linked immunosorbent assay; HPLC, high performance liquid chromatography; PEN, pentosidine; PINP, procollagen type I amino-terminal propeptide; ROC, Receiver Operating Characteristics; βCTX, type 1 collagen cross-linked C-telopeptide
